# Retraction: Chemosensitizing indomethacin-conjugated dextran-based micelles for effective delivery of paclitaxel in resistant breast cancer therapy

**DOI:** 10.1371/journal.pone.0348849

**Published:** 2026-05-07

**Authors:** The PLOS One Editors

The *PLOS One* Editors retract this article [1] due to concerns about authorship, reliability of the reported research, provenance of the reported work, and animal ethics. We regret that the issues were not identified prior to the article’s publication.

All authors either did not respond directly or could not be reached.
